# Educational and employment outcomes associated with childhood traumatic brain injury in Scotland: A population-based record-linkage cohort study

**DOI:** 10.23889/ijpds.v8i2.2327

**Published:** 2023-09-14

**Authors:** Meghan Visnick, Jill Pell, Daniel Mackay, David Clark, Albert King, Michael Fleming

**Affiliations:** 1 University of Glasgow, Glasgow, United Kingdom; 2 Public Health Scotland, Edinburgh, United Kingdom; 3 Scottish Government, Edinburgh, United Kingdom

## Objectives

Traumatic brain injury (TBI) is a leading cause of death and disability among young children and adolescents and the effects can be lifelong and wide-reaching. This study aimed to compare the educational and employment outcomes of Scottish schoolchildren previously hospitalised for TBI with their peers.

## Methods

A retrospective, record-linkage population cohort study was conducted using linkage of health and education administrative records. The cohort comprised all 766,244 singleton children born in Scotland and aged between 4 and 18 years who attended Scottish schools at some point between 2009 and 2013. Outcomes included special educational need (SEN), examination attainment, school absence and exclusion, and unemployment. Logistic regression models and generalised estimating equation (GEE) models were run unadjusted and then adjusted for sociodemographic and maternity confounders.

## Results

Of the 766,244 children in the cohort, 4,788 (0.6%) had a history of hospitalisation for TBI. Following adjustment for potential confounders, previous TBI was associated with SEN (OR 1.28, CI 1.18 to 1.39, p < 0.001), absenteeism (IRR 1.09, CI 1.06 to 1.12, p < 0.001), exclusion (IRR 1.33, CI 1.15 to 1.55, p < 0.001), and low attainment (OR 1.30, CI 1.11 to 1.51, p < 0.001). There was no significant association with unemployment 6 months after leaving school (OR 1.03, CI 0.92 to 1.16, p = 0.61). Excluding hospitalisations coded as concussion strengthened the associations.

## Conclusion

Childhood TBI, sufficiently severe to warrant hospitalisation, was associated with a range of adverse educational outcomes. These findings reinforce the importance of preventing TBI where possible. Where not possible, children with a history of TBI should be supported to minimise the adverse impacts on their education.

